# Novel Pharmaceutical and Nutraceutical-Based Approaches for Cardiovascular Diseases Prevention Targeting Atherogenic Small Dense LDL

**DOI:** 10.3390/pharmaceutics14040825

**Published:** 2022-04-09

**Authors:** Jelena Vekic, Aleksandra Zeljkovic, Aleksandra Stefanovic, Natasa Bogavac-Stanojevic, Ioannis Ilias, José Silva-Nunes, Anca Pantea Stoian, Andrej Janez, Manfredi Rizzo

**Affiliations:** 1 Department of Medical Biochemistry, University of Belgrade-Faculty of Pharmacy, 11000 Belgrade, Serbia; jelena.vekic@pharmacy.bg.ac.rs (J.V.); aleksandra.zeljkovic@pharmacy.bg.ac.rs (A.Z.); aleksandra.stefanovic@pharmacy.bg.ac.rs (A.S.); natasa.bogavac@pharmacy.bg.ac.rs (N.B.-S.); 2 Department of Endocrinology, Diabetes and Metabolism, Elena Venizelou Hospital, 11521 Athens, Greece; iiliasmd@yahoo.com; 3 Department of Endocrinology, Diabetes and Metabolism, Centro Hospitalar Universitário Lisboa Central, 1069-166 Lisbon, Portugal; silva.nunes@nms.unl.pt; 4 Nova Medical School, Faculdade de Ciencias Medicas, Universidade Nova de Lisboa, 1169-056 Lisbon, Portugal; 5 Health and Technology Research Center (HTRC), Escola Superior de Tecnologia da Saude de Lisboa, 1990-096 Lisbon, Portugal; 6 Faculty of Medicine, Diabetes, Nutrition and Metabolic Diseases, Carol Davila University, 050474 Bucharest, Romania; ancastoian@yahoo.com; 7 Department of Endocrinology, Diabetes and Metabolic Diseases, University Medical Centre, University of Ljubljana, 1000 Ljubljana, Slovenia; andrej.janez@kclj.si; 8 Department of Health Promotion, Mother and Child Care, Internal Medicine and Medical Specialties, University of Palermo, 90100 Palermo, Italy

**Keywords:** lipids, lipoproteins, small dense LDL, cholesterol, prevention, therapy

## Abstract

Compelling evidence supports the causative link between increased levels of low-density lipoprotein cholesterol (LDL-C) and atherosclerotic cardiovascular disease (CVD) development. For that reason, the principal aim of primary and secondary cardiovascular prevention is to reach and sustain recommended LDL-C goals. Although there is a considerable body of evidence that shows that lowering LDL-C levels is directly associated with CVD risk reduction, recent data shows that the majority of patients across Europe cannot achieve their LDL-C targets. In attempting to address this matter, a new overarching concept of a lipid-lowering approach, comprising of even more intensive, much earlier and longer intervention to reduce LDL-C level, was recently proposed for high-risk patients. Another important concern is the residual risk for recurrent cardiovascular events despite optimal LDL-C reduction, suggesting that novel lipid biomarkers should also be considered as potential therapeutic targets. Among them, small dense LDL particles (sdLDL) seem to have the most significant potential for therapeutic modulation. This paper discusses the potential of traditional and emerging lipid-lowering approaches for cardiovascular prevention by targeting sdLDL particles.

## 1. Introduction

The pathogenesis of atherosclerotic cardiovascular disease (CVD) involves the interplay of multiple pathophysiological processes, exposing a complex network of emerging risk factors and therapeutic targets. Nevertheless, elevated low-density lipoprotein-cholesterol (LDL-C) has been recognized as one of the few risk factors with a fundamental causative role in CVD [1]. However, today, it is widely accepted that routine quantification of LDL-C level does not provide an insight into the quality of circulating LDL pool, which is composed of a mixture of particles, differing in lipid content, density, size, electric charge, and potential proatherogenic properties [2]. Among them, the assessment of small dense LDL (sdLDL) particles is of particular importance since it is established that they have the greatest atherogenic potential [2]. Nevertheless, the complexity of techniques for measuring sdLDL seems to be the major obstacle for wider application of this biomarker in clinical practice [3]. Similarly, although statins remain a cornerstone of current lipid-lowering therapy [1], a clear benefit of introducing innovative therapeutic approaches [4] and nutraceuticals [5] in the lipid management of high-risk patients should not be neglected.

Small dense LDL particles, being profoundly involved in atherosclerosis development, are highly important as a target for modern pharmacological and non-pharmacological therapy. In recent years, novel therapeutics with great potential for sdLDL reduction, beyond of that achieved by traditional lipid-lowering medications, have been approved. Furthermore, the results of intervention studies using nutraceuticals have pointed to several prospective candidates with a favorable impact on these atherogenic particles. This paper will review the effects of current and emerging therapeutic options for the modulation of sdLDL particles.

## 2. Small Dense LDL and Cardiovascular Risk

One of the main clinically important consequences of plasma LDL heterogeneity is that a considerable proportion of patients might develop CVD despite optimal LDL-C concentrations. This phenomenon is attributed to the differences in cholesterol content within LDL particles among individuals exhibiting similar LDL-C levels [6]. Indeed, numerous studies have confirmed that smaller LDL particle size, increased sdLDL proportion, and/or elevated cholesterol concentration carried by sdLDL (sdLDL-C) are associated with the onset of CVD, independently of LDL-C level [7,8,9]. These data clearly indicate the superiority of LDL quality over LDL quantity in CVD risk prediction. Of note, it has been suggested that electric charge can also affect the atherogenic properties of LDL. Namely, it has been demonstrated that more electronegative LDL subfractions are present in a higher extent in the plasma of hypercholesterolemic subjects [10]. Moreover, previous studies have shown that these particles induce apoptosis and inhibit the differentiation of endothelial cells [10,11]. Recent evidence pointed toward the proatherogenic role of electronegative LDL, which is accomplished through the promotion of inflammation, macrophage differentiation, and triglyceride (TG) accumulation in lipid droplets [12]. However, as recently reviewed by Rivas-Urbina et al. [13], a full spectrum of biological roles of electronegative LDL in various pathological conditions remains to be elucidated. Interestingly, it has been shown that the amount of electronegative LDL subfractions determined by capillary isotachophoresis correlates negatively with LDL particle size, thus suggesting that electronegative LDL is accumulated in the sdLDL fraction [14]. In addition, Zhang et al. [15] demonstrated that rosuvastatin treatment significantly reduced the electronegative LDL contained in both large and sdLDL, thereby implying another pleiotropic effect of statins. Bearing in mind an obvious similarity in the biological effects of electronegative LDL and sdLDL particles, the question of their association and potential synergistic activity during atherosclerosis development deserves further investigation.

Insulin resistance is a well-studied metabolic alteration that serves as a driving force for increased sdLDL formation. In brief, an insulin-resistant state favors production and simultaneously delays catabolism of very-low density lipoprotein (VLDL), which results in hypertriglyceridemia [16]. Further activation of cholesterol-ester transfer protein (CETP) stimulates the exchange of triglycerides and cholesterol-esters (CE) between VLDL and LDL particles. The cascade of events culminates through the hydrolysis of TG-rich LDL particles by hepatic lipase, which converts them into sdLDL [17]. Of note, high-density lipoproteins (HDL) also undergo similar modification, which ultimately leads to a reduction in HDL-C level [3]. Therefore, insulin-resistant individuals are usually characterized by a specific form of atherogenic dyslipidemia, comprising elevated TG, low HDL-C level, and preponderance of sdLDL particles [18].

**Figure 1:**
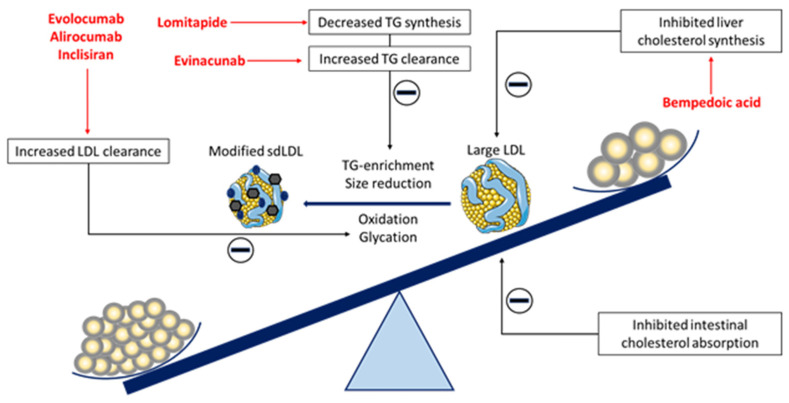
Therapeutic approaches for the reduction in sdLDL particles.

A crucial step in atherosclerotic plaque formation is cholesterol accumulation in macrophages and their transition to lipid-rich foam cells in the arterial wall. SdLDL particles are recognized as the main origin of deposited cholesterol in atherosclerotic plaque. Compared to larger LDL subfractions, sdLDL particles possess considerably higher atherogenic capacity due to delayed clearance from plasma and easier accumulation in the subendothelial space [2]. In addition, sdLDL particles are more prone to structural modifications, which further increase their proaterogenic properties. Adverse modifications of sdLDL particles include oxidation, desialylation, glycation, and alterations of protein components [19]. Clinically, the most important consequence of sdLDL accumulation in plasma and subendothelial space is their subsequent transformation into oxidized LDL particles (oxLDL). These particles have a central role in foam cell formation, but also possess potent biological effects in terms of promoting endothelial dysfunction, inflammation, oxidative stress, cell proliferation, and thrombosis, thereby further contributing to plaque progression and destabilization [20]. Hence, several categories of patients with this specific metabolic disorder, intimately associated with increased CVD risk, could benefit from sdLDL measurement and therapeutic modulation (Figure 1).

To summarize, screening for sdLDL should be advised to a wide range of high-risk subjects including those with multiple risk factors, particularly metabolic syndrome, patients with type 2 diabetes mellitus, but also patients who need secondary prevention, in attempting to reduce the residual risk. Although lipid guidelines, as the framework for CVD risk assessment, recommend standard lipid screening, advanced lipid testing might identify individuals with hidden cardiovascular risk by assessing the alterations of a wide range of lipid biomarkers. In this manner, assessment of LDL heterogeneity including sdLDL or LDL particle number (LDL-P) can be achieved by employing ultracentrifugation, gradient gel electrophoresis, HPLC, NMR, and ion mobility techniques [21]. Aside from these sophisticated methods, homogeneous assays for cholesterol content within sdLDL (sdLDL-C) have become available in routine laboratories, thus offering the possibility of a personalized approach in CVD prevention as well as of therapeutic individualization.

## 3. The Effect of Novel Lipid-Lowering Therapies on sdLDL Particles

Currently, the effects of novel lipid-lowering therapies on sdLDL reduction have been less explored, mainly because these medications were only recently approved. Therefore, this section will discuss their potential beneficial effects on sdLDL, based on the pharmacological mechanisms and available data gathered from observational studies and clinical trials.

### 3.1. PCSK9-Targeted Therapies

Proprotein convertase subtilisin/kexin type 9 (PCSK9) is a serine protease that enhances the degradation of hepatic LDL receptors, thus abolishing the clearance of LDL particles by the liver [22]. So far, two innovative approaches for targeting PCSK9 including inhibition of its activity or synthesis have emerged as effective and safe interventions for LDL-C lowering and cardiovascular prevention. In particular, two monoclonal anti-PCSK9 antibodies, evolocumab and alirocumab, are able to bind PCSK9 in plasma and antagonize its action while inclisiran, a small interfering ribonucleic acid (siRNA), prevents the translation of PCSK9 mRNA [4]. Both strategies aim to re-establish cholesterol homeostasis by increasing the expression of LDL receptors, which will consequently promote the clearance of LDL particles by the liver. Convincing data from the FOURIER [23] and ODYSSEY OUTCOMES [24] trials demonstrated substantial LDL-C level reduction by 50% with evolocumab and alirocumab, respectively. At present, PCSK9 inhibitors are recommended for patients with familial hypercholesterolemia and secondary prevention of high-risk patients who fail to reach the LDL-target despite optimal treatment or those intolerant to statins [4].

Regarding inclisiran, data from phase III trials (Orion 10 and 11) showed that LDL-C level reduction of 50% can be achieved by two doses per year [25,26]. In late 2020, inclisiran was authorized in the EU for the treatment of patients with primary hypercholesterolemia and the secondary prevention for patients who require additional LDL-C lowering [27]. Toward the end of 2021, inclisiran was also approved by FDA.

Several observational studies have shown a positive correlation of plasma PCSK9 and sdLDL levels in high-risk patients [28,29]. Thus, targeting PCSK9 might also be a promising strategy to control the level of circulating sdLDL particles. Analysis of the data from the DESCARTES trial showed that addition of evolocumab to statin therapy in patients with hyperlipidema significantly reduced concentrations of TG-rich lipoproteins and both large and small LDL particles [29]. Similarly, a post hoc analysis of phase II alirocumab trials showed a significant reduction in cholesterol content in all atherogenic lipoproteins including small dense LDL subclasses [30]. In addition, recent studies in patients with familial hypercholesterolemia suggest that beneficial effects of PCSK9 inhibitor therapy on sdLDL particles are associated with an improvement in endothelial function [31] and carotid stiffness [32]. While exploring the potential benefit of PCSK9 targeting for sdLDL reduction, one should not neglect that inclisiran is also efficient in reducing atherogenic lipoproteins (i.e., non-HDL-cholesterol and Lp(a) levels) [26,33,34]. However, the effects on sdLDL are yet to be determined.

### 3.2. Bempedoic Acid

An innovative approach of LDL-C lowering by bempedoic acid offers an additional option for targeting sdLDL particles. Namely, bempedoic acid attenuates the activity of ATP-citrate lyase (ACL), an enzyme responsible for the generation of acetyl-coenzyme A, the central building unit for lipogenesis and steroidogenesis [35]. Since bempedoic acid inhibits cholesterol synthesis at an earlier point than HMG-CoA reductase, it is useful as an add-on therapy to statins and/or ezetimibe. By inhibiting cholesterol synthesis, bempedoic acid causes the upregulation of hepatic LDL receptors and consequent clearance of LDL particles, thus lowering plasma LDL-C levels alone by 15–25%, while in combination with ezetimibe, more than 30% [36]. Bempedoic acid was authorized in the U.S. and the EU for primary prevention of patients with heterozygous familial hypercholesterolemia and for secondary prevention of CVD patients who require additional lowering of the LDL-C level [37].

Data from clinical trials showed that the beneficial effects of bempedoic acid are also reflected in the improvement in other lipid status parameters such as non-HDL-C and apolipoprotein B (apo B), but also on the level of high-sensitivity C-reactive protein (hsCRP), a marker of subclinical inflammation [38]. A meta-analysis of phase II and phase III trials showed that bempedoic acid significantly reduced the LDL particle number (LDL-P), but had no effects on VLDL particle number and TG level [39]. It also has a beneficial effect on serum glucose level by activating liver AMP-kinase [40]. Notably, in a recent analysis of pooled data from phase III trials, the use of bempedoic acid was not associated with the risk for diabetes onset or worsening metabolic control in patients with diabetes [41]. Hence, bempedoic acid seems to be safe for insulin-resistant subjects. This specific category of patients would also be expected to benefit the most from sdLDL reduction by bempedoic acid, but this effect remains to be tested in the future.

### 3.3. Lomitapide

Lomitapide is a small molecule that binds and inhibits microsomal triglyceride transfer protein (MTP) in the liver and intestine. Since MTP facilitates the transfer of TG, phospholipids, and CE to apoB during the assembling processes of chylomicrons and VLDL particles [42], inhibition of its activity reduces the levels of TG-rich lipoproteins in plasma. One of the first MTP inhibitors, tested in the animal model of human homozygous familial hypercholesterolemia, demonstrated a reduction in plasma apoB-containing lipoproteins without alterations of liver enzymes [43]. However, since inhibition of MTP induced accumulation of TG in hepatic and intestinal cells, further research was abandoned due to the possible adverse effects [44]. Lomitapide was evaluated in a phase III study in homozygous familial hypercholesterolemia patients with ongoing lipid-lowering therapy and apheresis. It was found that LDL-C level decrease was dose-dependent and that most patients achieved a LDL-C reduction of more than 50% [45]. The efficacy of lomitapide was confirmed by the results of a long-term extension study [46] as well as by another clinical study [47] and real-world data [48]. The treatment also reduced TG and non-HDL-C levels [46]. However, high doses were associated with adverse effects such as steatorrhea, gastrointestinal symptoms, or liver steatosis. At present, lomitapide has been approved only for the management of homozygous familial hypercholesterolemia [49].

Despite beneficial effects on serum TG level, there is no convincing data to conclude whether lomitapide could be considered for the management of atherogenic dyslipidemia. Based on the data from a phase III clinical study, lomitapide treatment was associated with a moderate decrease in HDL-C levels [45]. However, Yahya et al. [50] showed that such a reduction in HDL-C was followed by the shift of the HDL subclass distribution toward more extensive, more buoyant HDL 2 particles, without significant impact on cholesterol efflux capacity (i.e., HDL functionality). Currently, no available data have demonstrated the effects of lomitapide on the sdLDL particles. Observational data suggest that a common polymorphism −492G/T within the promoter of the *MTP* gene is not associated with variations in LDL size and subclass distribution [51]. However, these findings do not exclude targeting of MTP as a potential strategy for the modulation of sdLDL, and the potential effects of lomitapide need to be evaluated in further studies.

### 3.4. Evinacumab

Angiopoietin-like protein 3 (ANGPTL3) is a member of the ANGPTL family of proteins with numerous functions in lipid metabolism, inflammation, glucose homeostasis, and cancer [52]. ANGPTL3 primarily regulates lipid metabolism by modulating the availability of TG for adipose, heart, and skeletal muscles throughout the inhibition of lipoprotein (LPL) and endothelial lipase (EL) [53]. The deficiency of ANGPTL3 enhances the clearance of TG-rich lipoproteins and carriers of *ANGPTL3* mutations have lower plasma TG, LDL-C and HDL-C levels, and reduced CVD risk [54]. Thus, ANGPTL3 is recognized as an emerging therapeutic target for dyslipidemia.

Evinacumab is a fully human monoclonal antibody directed against ANGPTL3. It binds the N-terminal domain of ANGPTL3 and abolishes the inhibition of LPL and EL [55]. Evinacumab demonstrated a dose-dependent reduction in LDL-C, TG, non-HDL-C, HDL-C, total cholesterol, apoB, and Lp(a) levels in a randomized, double-blind, placebo-controlled trial including subjects with moderately elevated LDL-C levels [56]. In the phase III ELIPSE HoFH study, evinacumab administration was associated with a reduction in LDL-C and TG levels by 49% and 50%, respectively [57]. A proof-of-concept study showed that the addition of evinacumab to standard lipid-lowering therapy of familial hypercholesterolemia further reduced LDL-C levels [58]. The effect on LDL-C level reduction is likely to be mediated by an increased catabolic rate of apoB within the LDL particles and their precursors [59]. Evinacumab has been authorized for the treatment of homozygous familial hypercholesterolemia by FDA and EMA since 2021 [60].

Recent studies in apparently healthy subjects [61] and patients with diabetes [62] have reported a positive association between increased plasma ANGPTL3 and sdLDL particles. These findings suggest possible beneficial effects of ANGPTL3 inhibition on sdLDL, which should be verified in the future. Aside from evinacumab, two additional approaches for the silencing of ANGPTL3 synthesis by antisense oligonucleotides (ANGPTL3-RLX) and siRNA (ARO-ANG3) are currently being investigated in clinical trials. Furthermore, Fukami et al. [63] recently developed a peptide vaccine for the targeting of ANGPTL3 and demonstrated a reduction in sdLDL-C level in a preclinical study, offering a promising new approach to the treatment of atherogenic dyslipidemia.

## 4. The Effect of Nutraceuticals on sdLDL Particles

Dietary supplements and functional food are recommended for lowering LDL-C concentrations in individuals with high cholesterol concentrations and intermediate or low cardiovascular risk who are not candidates for pharmacotherapy as well as in patients with high and very high cardiovascular risk but with inadequate response to pharmacotherapy [1]. The LDL-C lowering effects of nutraceuticals rise from their capability to modulate basic processes of exogenous cholesterol absorption, endogenous cholesterol synthesis, its metabolism, and elimination (Figure 1). By affecting these and several other processes within lipoprotein metabolism, nutraceuticals can also modulate sdLDL particles. However, scientific research still needs to fully confirm clinical evidence of the nutraceuticals’ efficacy and evaluate its safety and tolerability.

### 4.1. Phytosterols

Primary food sources of phytosterols are vegetable oil, vegetable margarine, cereal products, vegetables, nuts, and legumes. Dominant phytosterols from food are β-sitosterol, campesterol, stigmasterol, and campestanol [64]. Approximate daily intake is between 250 mg/day in Northern Europe to 500 mg/day in Mediterranean countries [1]. Phytosterols directly compete with exogenous intestinal cholesterol for the Neimann-Pick C1-Like 1 (NPC1L1) transporter on enterocytes and thus reduce cholesterol absorption. A compensatory increase in hepatic LDL receptor expression follows diminished intestinal cholesterol uptake, which altogether results in a decrease in plasma LDL-C concentration [65]. Daily intake of 2 g of phytosterols can decrease the total cholesterol (TC) and LDL-C levels up to 10%, with little or no effects on HDL-C and TG concentrations [1,66]. Regarding the qualitative and quantitative characteristics of LDL particles, the results of previous studies showed that intake of phytosterols has positive effects [67,68]. In patients with metabolic syndrome, two months of supplementation with four g/day of phytosterols resulted in reduced TC, LDL-C, and sdLDL-C levels [69]. Furthermore, the results of Garoufi et al. [70] showed that daily consumption of 2 g of plant sterols decreased sdLDL-C levels in children with hypercholesterolemia. On the other hand, 12 weeks of supplementation with 2.6 g/day of phytosterol esters showed no effects on the proportion of LDL subclasses and mean LDL diameter in hypercholesterolemic adults who were not taking lipid-lowering therapy [70]. Nevertheless, recent scientific findings support the view that adequate daily phytosterol supplementation can lower cardiovascular risk in hypercholesterolemic individuals by reducing sdLDL particles [1]. Current European recommendations endorse a supplementation with more than two g/day of phytosterols in hypercholesterolemic individuals with low or intermediate cardiovascular risk and adults and children with familial hypercholesterolemia [1]. Daily phytosterol intake is also recommended for high- and very high-risk patients who fail to achieve recommended LDL-C goals with statin therapy [1]. Possible adverse effects of phytosterol supplementation are related to potential lower intestinal absorption of fat-soluble vitamins [71]. Notably, these nutraceuticals are not recommended for individuals with sitosterolemia, a metabolic disorder resulting from a mutation in genes that encode ABCG5/G8 transporters and subsequent retention of phytosterols [72].

### 4.2. Dietary Fiber

Dietary fiber, soluble and non-soluble, mainly found in vegetables, fruits, grains, and legumes, are nutraceuticals resistant to digestion in the small intestine with beneficial effects on cholesterol metabolism. The mechanisms of action of dietary fiber (pectin, β-glucans, psyllium) with respect to cholesterol metabolism are not completely resolved, but their main effect is attributed to the ability to boost the excretion of bile acids and fecal cholesterol [73,74]. In this way, dietary fiber indirectly modifies LDL receptor expression and enhances the clearance of LDL particles [75]. A systematic review and meta-analysis of randomized controlled trials showed that daily intake of approximately 3.5 g of β-glucan significantly reduced LDL-C concentration [76]. Shrestha et al. [77] showed that daily intake of a combination of psyllium and phytosterols reduced LDL-C concentration, but also reduced the medium and small LDL particles and increased LDL size. Current ESC/EAS guidelines recommend a daily intake of 3 to 10 g of dietary fiber to achieve a relevant reduction of 3–5% in LDL-C concentration [1].

### 4.3. Monacolin K

Being the main source of LDL-C in plasma, liver cholesterol synthesis also affects the amount of every LDL subclass. However, in contrast to the well-studied beneficial effects of pharmacological agents HMG-CoA reductase inhibitors on the size and distribution of LDL subclasses, only scarce evidence is available regarding natural products with similar activity. Red yeast rice’s major bioactive constituent monacolin K, also known as lovastatin, is renowned for its beneficial hypolipemic effects [78]. To the best of our knowledge, no studies have analyzed the effects of isolated monacolin K on sdLDL particles. However, a recent study of Galletti et al. [79] revealed a significant increase in LDL particle size in patients with metabolic syndrome after 24-week treatment with a combination of lipid-lowering nutraceuticals containing monacolin K, berberine, and policosanol. Administration of the same nutraceuticals reportedly caused a reduction in the relative proportion of sdLDL particles in a group of 30 patients with familial combined hyperlipidemia [80]. However, it should be appreciated that other components of the mentioned nutraceutical combination also exhibit hypocholesterolemic effects. Specifically, policosanol, a long-chained alcohol derived from various fruits, nuts, sugarcane etc., also acts as an inhibitor of cholesterol synthesis as well as a stimulator of LDL uptake and cholesterol excretion [81]. Similarly, berberine can enhance LDL uptake by upregulation of LDL receptors and inhibition of PCSK9 [82,83]. Therefore, it remains to be established how these nutraceuticals separately affect plasma sdLDL and whether they act synergistically when combined. Regarding the safety of the supplementation, monacolin K adverse effects mostly arise from its analogy with statins; possible rhabdomyolysis is the major concern. However, available evidence suggests that administration of 3–10 mg/day of monacolin K has minimal side effects, so it is generally considered safe [84].

### 4.4. Polyphenols

Flavonoids are among the best-studied polyphenols in terms of hypolipemic activity. However, their precise effects on the redistribution of lipoprotein particles are largely unexplored. However, there is evidence of the specific influence of several flavonoids on sdLDL particles. For example, it has been demonstrated that citrus flavonoids such as naringin and hesperidin exhibit lipid-lowering effects by inhibiting HMG-CoA reductase and acyl-CoA:cholesterol O-acyltransferase (ACAT) activities in rats [85]. Likewise, bergamot (*Citrus bergamia*) flavonoids reportedly possess statin-like effects [5]. It has also been shown that the administration of bergamot flavonoid extract (neoeriocitrin, neohesperidin and naringin) for six months resulted in a significant increase in large, alongside a concomitant decrease in small LDL subclass proportions, in subjects with hypercholesterolemia [86]. Similar results were reported for the soluble derivative of hesperidin, which is an abundant flavonoid in citrus fruit peel. Namely, it was demonstrated that the 24-week long administration of 500 mg/day of glucosyl-hesperidin to hypertriglyceridemic patients caused an increase in LDL particle size [87]. Furthermore, in general, citrus flavonoids have a good safety profile and minimal side effects [88], so they could be promising agents in ameliorating LDL subclass distribution, and can ultimately be used for the prevention and treatment of cardiovascular disease [88].

Soy isoflavones such as genistein are capable of the downregulation of HMG-CoA activity [89]. Accordingly, a meta-analysis showed that soy isoflavones significantly reduce TC and LDL-C concentrations [90]. In contrast, any beneficial influence of isolated soy isoflavone administration on sdLDL particles was not proven in postmenopausal women [91]. It should also be appreciated that several concerns were raised concerning the safety of soy isoflavones. Namely, due to their estrogen-like effects, it is necessary to clarify whether using these compounds could be associated with an increased risk for specific types of cancer [92].

Other polyphenols also affect cholesterol synthesis. It has been shown that curcuminoids, bioactive polyphenolic compounds of turmeric (*Curcuma longa*), cause a decrease in the enzymatic activity of HMG-CoA reductase, among other effects [93,94]. Although the impact of curcuminoids on serum lipid profile has been confirmed [95], Moohebati et al. [96] reported that short-term (four weeks) supplementation with 1 g/day of curcuminoids did not provide any changes in sdLDL-C. Similarly, Panahi et al. [97] found no differences in sdLDL-C after eight weeks of prolonged curcuminoid supplementation in patients with metabolic syndrome.

### 4.5. Omega-3 Long-Chain Polyunsaturated Fatty Acids

It has long been documented that omega-3 long-chain polyunsaturated fatty acids (n-3 LCPUFA) can significantly modulate the lipid profile, mainly by reducing TG levels [5]. The two most intensively studied n-3 LCPUFA, relevant to human health are eicosapentaenoic acid (EPA) and docosahexaenoic acid (DHA). Many previous studies have explored the influence of n-3 LCPUFA administration on sdLDL. Bearing in mind the contribution of hypertriglyceridemia in sdLDL genesis, it was reasonable to assume that n-3 LCPUFA, by their TG-lowering activity, can indirectly affect sdLDL particles. Indeed, it has been shown that DHA supplementation (3 g/day) for 90 days improved LDL subclass distribution in both fasting and postprandial plasma of patients with hypertriglyceridemia [98]. Similar results were obtained after six week long administration of 1.52 g/day of DHA in subjects with decreased HDL-C [99]. Regarding EPA, Satoh et al. [100] demonstrated that purified EPA (1.8 g/day for three months) provoked a decrease in the proportion of sdLDL-C and sdLDL particles as well as in CETP activity. Moreover, a recent in vitro study showed that EPA is highly potent in preventing the oxidation of sdLDL [101].

The combined effects of EPA and DHA have been examined in various clinical studies. Thus, it has been shown that the concentration of large LDL particles increased and the concentration of sdLDL decreased in type 2 diabetes patients after 12 weeks of supplementation with 4 g/day of n-3 LCPUFA, in parallel with their standard treatment [102]. Similarly, as reported by Agouridis et al. [103], combined therapy of low-dose rosuvastatin and n-3 LCPUFA (2 g/day) was equally effective in raising LDL particle size, along with high-dose rosuvastatin in patients with mixed dyslipidemia and metabolic syndrome. Considering the significant side effects of high-dose statins, these results might be of interest for the individualization of hypolipemic therapy in subjects with an inadequate response to statins. In addition, a recent randomized, open-label phase 4 study demonstrated that 8-week treatment with n-3 LCPUFA (2 g/twice-daily) in combination with statins yielded a significant increase in LDL particle size in dyslipidemic patients [104]. Interestingly, 3-month administration of 1.7 g/day of n-3 LCPUFA did not cause any changes in sdLDL percentage in patients with end-stage renal disease [105].

Other n-3 PUFAs might also be of interest regarding their putative effects on atherogenic dyslipidemia. Kawakami et al. [106] demonstrated that 12-week supplementation with flaxseed oil, as an abundant source of alpha-linolenic acid (ALA), is associated with a significant decrease in sdLDL-C concentration in healthy adult Japanese men. In contrast, Wilkinson et al. [107] demonstrated that 12-week dietary intake of flaxseed oil did not cause significant changes in LDL subclass distribution when compared to the supplementation with fish oil in healthy male adults. Interestingly, Tuccinardi et al. [108] demonstrated that walnut consumption decreased the concentration of sdLDL particles in obese individuals. Walnuts (*Juglans regia*) are rich in polyphenols and fiber, but especially in ALA. However, it should be noted that sample size in the majority of these studies was rather small, so larger studies are needed, either to confirm or to rule out any contribution of ALA to the amelioration of LDL subfraction profile.

A meta-analysis of 21 randomized controlled trials [109] has shown that n-3 LCPUFA containing products are well tolerated, although several side effects are present including fishy taste, dermatological and gastrointestinal problems as well as alterations of biochemical parameters. However, these adverse effects were mild in general. Recently, it has been suggested that the genetic background might be responsible for individual susceptibility to adverse reactions after supplementation with n-3 LCPUFA [110] and this could have important consequences in terms of an individualized approach during the administration of these nutraceuticals.

### 4.6. Other Nutriceuticals

Prickly pear (*Opuntia ficus indica*) is a plant from the Cactaceae family that is distributed worldwide including in the Americas, Africa, Australia, and the Mediterranean. The prickly pear pulp, peel, seeds, and cladodes are rich in PUFA, phytosterols, and phenolic compounds, and this plant is traditionally recognized by its beneficial health effects [111]. It has been shown that a 4-week regular intake of pasta containing Opuntia ficus extract decreased the percentage of sdLDL particles in subjects with at least one criterion for metabolic syndrome fulfilled [112]. These findings advocate for a wider use of prickly pear as part of a nutraceutical approach to dyslipidemia.

Oolong tea is a moderately fermented extract of the leaves of *Camellia sinensis*, which is a mixture of polyphenolic compounds, catechins, and their polymeric derivatives theaflavins and arubigins [113]. The main oolong tea antiatherogenic effect is most likely a reduction in TC and LDL-C concentrations. Proposed mechanisms of action involve the polyphenols’ antioxidative properties, activation of the AMPK signaling pathway, inhibition of HMG-CoA reductase, interaction with cholesterol absorption, and inhibition of bile acid reabsorption [114,115]. In addition, Shimada et al. [116] showed that oolong tea consumption affected the atherosclerosis progression in patients with coronary artery disease by improving the lipid profile and slightly increasing the LDL particle size.

It has been found that biologically active soy proteins and peptides could be involved in the regulation of LDL receptor activity [117], reduction in intestinal cholesterol absorption [118], cholesterol biosynthesis rate, and increase in bile salt fecal excretion [119]. Clinical evidence for these effects of soy proteins has been recently published in a meta-analysis of 46 studies [120]. The authors concluded that soy proteins significantly reduced TC and LDL-C concentrations in adults and supported plant protein dietary intake [120]. Furthermore, Desroches et al. [121] showed that soy protein consumption shifted LDL particle distribution toward a less atherogenic pattern in hypercholesterolemic individuals older than 50 years.

Although not nutraceuticals in strict terms, probiotics can modify the gut microbiome, thereby altering the bioavailability of dietary compounds and affecting the entire energy metabolism. For these reasons, the impact of probiotics and the gut microbiome on metabolic changes associated with obesity has been the focus of contemporary research. Several mechanisms have been proposed to explain the hypocholesterolemic effects of probiotics: decreased absorption of intestinal cholesterol through its precipitation with deconjugated bile salts; assimilation of cholesterol by microbial cells; its metabolic transformation to coprostanol; or decreased expression of cholesterol transporter in the enterocyte membrane [122]. In line with this, the beneficial effects of probiotics on LDL-C have been reported [123,124], although not in all studies [125]. A recent study by Michael et al. [126] analyzed the influence of lactobacilli and bifidobacteria supplementation (50 billion CFU/day) for six months in overweight and obese individuals. Apart from a significant decrease in body weight, hypercholesterolemic patients also experienced a reduction in sdLDL-C following supplementation, which is an interesting finding that should be further explored. The use of probiotics is generally considered safe, but one should not neglect that adverse effects have been reported in vulnerable populations (e.g., infants, immunocompromised patients) [127]. Thus, further investigations are needed to fully evaluate the possible benefits of probiotics in controlling lipid profile abnormalities.

## 5. Conclusions

Even though sdLDL has been long recognized as an essential feature of atherosclerosis development, it is still neither a part of the routine assessment of CVD risk, nor a specific therapeutic target. At present, commercially available electrophoretic systems such as Lipoprint represent the most reliable routinely applicable method for the assessment of sdLDL levels [2]. In addition to sdLDL, this method provides an insight into the entire spectrum of LDL subclasses in plasma and is more economical than other methodologies [2]. Regarding the measurement of sdLDL alone, automated homogenous enzymatic assays for sdLDL-C concentration determination are the most readily available for medical laboratories, and therefore could be recommended as an optimal and feasible methodology for assessing sdLDL in clinical practice. Numerous investigations have demonstrated that both conventional pharmacological treatment and novel therapeutic approaches designed to affect specific aspects of lipid metabolism also target sdLDL particles. In addition, there is a plethora of evidence that nutraceuticals, besides their confirmed effects on LDL-C, can also modify a qualitative aspect of these particles. However, additional large-scale clinical trials are needed to fully evaluate the capabilities of various pharmacological and non-pharmacological approaches to improve LDL quality by reducing the sdLDL content.

Bearing in mind that genetic factors have recently been shown to contribute to the effectiveness of pharmaceutical approaches by targeting LDL, these factors should also be considered in the context of effective cardiovascular prevention. Namely, it is now firmly established that numerous genetic variants may cause inter-individual variations in response to lipid-lowering therapy [128]. So far, ample evidence is available confirming the association between the efficacy of statin therapy and certain single nucleotide polymorphisms of the genes encoding proteins that participate in cholesterol synthesis and removal from plasma such as HMG-CoA reductase, LDL-receptor, and PCSK9, but also apolipoprotein E. Investigators of the Prospective Study of Pravastatin in the Elderly at Risk (PROSPER) reported that polymorphism in the 3′-untranslated region (UTR) of the LDL-receptor gene contributed to different therapeutic response to pravastatin [129], while researchers of the Cholesterol and Pharmacogenetics (CAP) trial showed that a common haplotype within 3′-UTR of the LDL-receptor gene is associated with reduced response to simvastatin [130]. In the same study, the presence of both LDL-receptor and HMG-CoA reductase gene polymorphisms was associated with an additionally attenuated lipid-lowering effect of simvastatin [130]. The results of the Justification for the Use of Statins in Prevention: an Intervention Trial Evaluating Rosuvastatin (JUPITER) trial suggested that certain genetic variants of PCSK9 are also able to modulate response to statin therapy [131]. In line with the previous, Feng et al. [132] recently demonstrated a more favorable response to statins in the carriers of loss-of-function variants of the *PCSK9* gene. Regarding apolipoprotein E gene polymorphism, it was shown that the carriers of ε_2_ allele have more favorable response to atorvastatin [133], in contrast to the carriers of the ε_4_ allele [134]. Genetic variations of PCSK9 have also been proposed to affect the response to PCSK9 inhibitor therapy observed in clinical trials [135], but currently there is no available data to confirm this assumption. Overall, genetic testing could be useful for the identification of high-risk patients that cannot achieve therapeutic LDL-C goals. According to available evidence, the presence of mutations responsible for severe defects in the LDL-receptor and/or gain-of-function mutations of the *PCSK9* gene might suggest an unsatisfactory response to statin therapy. Given the fact that the latest guidelines recommend the addition of emerging lipid-lowering agents to statin therapy in patients requiring further LDL-C lowering, pharmacogenomic studies with combined therapy are highly welcomed. These data would hopefully pave the way for a personalized approach toward more efficient targeting of sdLDL.

## Data Availability

Not applicable.
